# Characterization of *Bacillus anthracis* Persistence In Vivo

**DOI:** 10.1371/journal.pone.0066177

**Published:** 2013-06-04

**Authors:** Sarah A. Jenkins, Yi Xu

**Affiliations:** Center for Infectious and Inflammatory Diseases, Institute of Biosciences and Technology, Texas A&M Health Science Center, Houston, Texas, United States of America; Loyola University Medical Center, United States of America

## Abstract

Pulmonary exposure to *Bacillus anthracis* spores initiates inhalational anthrax, a life-threatening infection. It is known that dormant spores can be recovered from the lungs of infected animals months after the initial spore exposure. Consequently, a 60-day course antibiotic treatment is recommended for exposed individuals. However, there has been little information regarding details or mechanisms of spore persistence *in vivo*. In this study, we investigated spore persistence in a mouse model. The results indicated that weeks after intranasal inoculation with *B. anthracis* spores, substantial amounts of spores could be recovered from the mouse lung. Moreover, spores of *B. anthracis* were significantly better at persisting in the lung than spores of a non-pathogenic *Bacillus subtilis* strain. The majority of *B. anthracis* spores in the lung were tightly associated with the lung tissue, as they could not be readily removed by lavage. Immunofluorescence staining of lung sections showed that spores associated with the alveolar and airway epithelium. Confocal analysis indicated that some of the spores were inside epithelial cells. This was further confirmed by differential immunofluorescence staining of lung cells harvested from the infected lungs, suggesting that association with lung epithelial cells may provide an advantage to spore persistence in the lung. There was no or very mild inflammation in the infected lungs. Furthermore, spores were present in the lung tissue as single spores rather than in clusters. We also showed that the anthrax toxins did not play a role in persistence. Together, the results suggest that *B. anthracis* spores have special properties that promote their persistence in the lung, and that there may be multiple mechanisms contributing to spore persistence.

## Introduction

Anthrax infections are caused by the entry of *Bacillus anthracis* spores into the host via the respiratory system, the gastrointestinal tract, and cuts or wounds in the skin. Among these three forms, inhalational anthrax has the highest lethality rate. One of the characteristic features of inhalational anthrax is the prolonged presence of spores in the lungs after an initial exposure. For example, dormant spores of *B. anthracis* were recovered from the lungs of non-human primates [1] and mice [2], [3] weeks or months post-exposure. It was also observed that sometimes there was a delayed onset of anthrax infections (*e.g.*, 58 days after exposure) in exposed animals, and the persistence of spores in the lung was thought to be responsible for the delay [4]. Based on these observations, the current duration of antibiotic treatment recommended for people with pulmonary exposure is 60 days.

Persistent colonization in a host by microbial pathogens has been a challenge for effective treatment. A variety of mechanisms contribute to pathogen persistence in the host. These mechanisms include suppression of host innate immune responses [5], modulation of complement activation [6], resistance to phagocytic killing [7], , adaptation to the intracellular environment or to the mucosal surfaces [9], [10], biofilm formation [11], and evasion of host adaptive immunity [12]. For *B. anthracis*, the mechanism for spore persistence in the lung has not been investigated but has been largely attributed to the dormant nature and the resilience of spores. However, recent findings raise the possibility that there may be other factors contributing to spore persistence. Russell *et al* showed that spores were found inside epithelial cells in the mouse lung within hours after exposure to spores and that internalized spores survived inside epithelial cells [13], [14]. Thus the intracellular environment of lung epithelial cells can potentially be a niche for spores to persist. There has also been evidence for biofilm formation by *B. anthracis*, *B. subtilis* and other related species and the presence of spores within the biofilm [15], [16], [17].

In this study, we investigated spore persistence in mice over a period of up to eight weeks. The spatial distribution of spores and their association with different host cells were examined. We also compared *B. anthracis* spores with spores from a non-pathogenic *Bacillus subtilis* strain. The contribution of uptake by host cells and anthrax toxins to spore persistence was also examined. The results suggest that *B. anthracis* spores possess special properties that promote their survival and persistence in the host.

## Results

### The lung is the primary site for *B. anthracis* spore persistence

**Figure 1:**
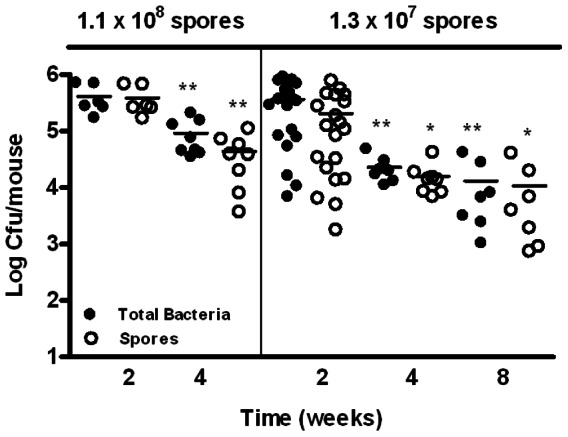
Bacterial and spore burden in the lungs of mice at 2, 4, and 8 weeks post-intranasal inoculation. Mice were inoculated i.n. with ∼1.1×10^8^ or ∼1.3×10^7^ spores/mouse. Lungs were harvested at 2, 4, and 8 weeks, homogenized, and dilution plated with or without heat treatment. The results were combined from at least two independent experiments. Closed circles represent total viable bacteria and open circles heat-resistant dormant spores. *, *p*<0.05; **, *p*<0.01; compared to respective total bacteria and spore titers at 2 weeks.

BALB/c mice were challenged intranasally (i.n.) with sub-lethal doses of spores of *B. anthracis* Sterne strain 7702 (pXO1^+^, pXO2^−^). BALB/c mice are generally more resistant to the Sterne strain [18] and therefore provided us a model to assay for persistence of spores where mice can survive weeks post initial infection. The presence of vegetative bacilli and spores in different organs was evaluated over a period of 8 weeks. The results indicated that substantial amounts of bacteria were recovered from the lungs of mice at 2, 4 and 8 weeks post-inoculation (Fig. 1 and Table 1). The majority of the bacteria recovered were heat-resistant dormant spores; however heat-sensitive vegetative bacilli were also detected at all three time points. Immunofluorescence staining of lung sections with antibodies specific for vegetative bacilli also showed positive staining (Supplemental figure S1), indicating that spore germination occurred in the lung although at a relatively low frequency. We observed decreases in the total bacteria and spore titers in the lung over the experimental period, suggesting a continuous host clearance process. The decreases were significantly sharper during the earlier weeks and were relatively moderate from 4 to 8 weeks (Fig. 1 and Table 1).

**Table 1:** Bacteria and spore burden in the lungs of mice.

Inoculum	Average Retained Dose^a^	Time	Total Bacteria/Spores^b^	*P* value^c^
(spores/mouse)	(cfu±SEM)	(weeks)	(cfu×10^4^/lung±SEM)	(vs. 2 weeks)
		2	37±6.5/20±5.0	-
1.3×10^7^	(1.5±0.1)×10^6^	4	2.3±0.4/1.6±0.4	<0.01/<0.05
		8	1.3±0.6/1.1±0.6	<0.01/<0.05
		2	41±9.7/39±9.2	-
1.1×10^8^	(2.6±0.7)×10^7^	4	9.2±2.3/4.4±1.3	<0.01/<0.01
		8	ND^d^	ND^d^

a Average retained dose was determined by harvesting the lungs 1 hour after intranasal inoculation and dilution plating the lung homogenates.

b The amounts of total bacteria and spores in the lung as described in the Figure 1 legend are expressed as the mean±SEM at 2, 4, or 8 weeks post-inoculation. Results are combined from two independent experiments.

c Statistical analysis was performed using the two-tailed Student's t-test. *, *p*<0.05; **, *p*<0.01; compared to respective total bacteria and spore titers at 2 weeks.

d Not determined.

**Figure 2:**
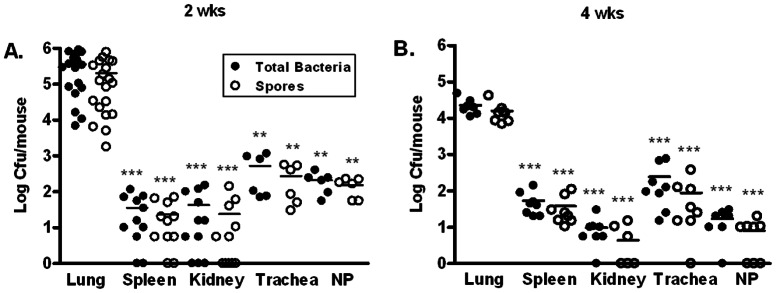
Bacterial and spore burden in various organs at 2 and 4 weeks post-inoculation. Mice were inoculated i.n. with ∼1.3×10^7^ spores/mouse. Bacterial burden in the lung, spleen, kidney, trachea, and nasopharynx (NP) at 2 (**A**) and 4 (**B**) weeks was determined as described in the Materials and Methods section. The results were combined from at least 2 independent experiments. Closed circles represent total viable bacteria and open circles heat-resistant dormant spores. **, *p*<0.01; ***, *p*<0.001; compared to respective total bacteria and spore titers in the lung.

To investigate if the lung was the major organ for spore persistence we examined the bacterial burden in other tissues. The bacterial titers in the spleen and kidney at 2 and 4 weeks were significantly lower (approximately 10^3^–10^4^ fold) than those in the lung (Fig. 2, A and B). Intranasal inoculation exposes the nasopharynx associated lymphoid tissue (NALT) to the spores. A previous report indicated that the NALT was among the first organs in which vegetative growth was observed following pulmonary exposure to spores [19]. We examined the bacterial burden in the nasopharynx as well as the trachea at 2 and 4 weeks post-inoculation. The results showed that both the total bacteria and spore titers in the trachea and the nasopharynx were significantly lower than those in the lung at both time points (Fig. 2, A and B). These results indicate that the lung is the primary site for spore persistence.

### *B. anthracis* has special properties that contribute to its ability to persist in mice

**Figure 3:**
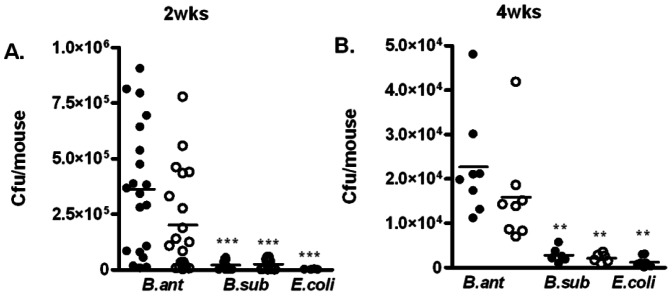
*B. anthracis* spores persisted in the lung significantly better than spores of *B. subtilis*. Mice were inoculated i.n. with 7702 spores (*B. ant*), PY79 spores (*B. sub*), or overnight cultures of HB101 (*E. coli*) at a dose of ∼1.3×10^7^ cfu/mouse. Lungs were harvested at 2 (**A**) and 4 (**B**) weeks post-inoculation, homogenized, and plated for total viable bacteria (closed circles) or heat-resistant dormant spores (open circles). The results were combined from at least 2 independent experiments. **, *p*<0.01; ***, *p*<0.001; compared to respective total bacteria and spore titers in *B. anthracis* infected lungs.

We next investigated whether the ability of *B. anthracis* spores to persist in the lung was due to special properties of *B. anthracis* or due to the dormant and resilient characteristics of spores in general. Therefore, we examined if spores from *Bacillus subtilis* could persist in the lung equally well as those from *B. anthracis*. Mice were inoculated i.n. with approximately 1.3×10^7^ 7702 or *B. subtilis* strain PY79 spores. Lungs were harvested at 2 and 4 weeks post-inoculation and evaluated for total viable bacteria and spores. At both time points, significantly less bacteria or spores were recovered from mice inoculated with *B. subtilis* compared to those inoculated with *B. anthracis* (Fig. 3, A and B), suggesting that *B. anthracis* spores are better at persisting in the mouse lung than *B. subtilis* spores. Mice were also inoculated with overnight cultures of *E. coli* HB101, a non-sporulating, non-pathogenic strain. These mice had significantly fewer bacteria in the lung than those infected with *B. anthracis* and *B. subtilis*, respectively (Fig. 3). We also calculated the spore burden in the lung as a percentage of the initial inoculum. At 2 weeks, the percentages were approximately 1.7%, 0.25% and 0.01% for mice infected with 7702, PY79 and *E. coli*, respectively. At 4 weeks, the percentages were approximately 0.15%, 0.02% and 0.01% for the three strains, respectively. These results suggest that in addition to dormancy and resilience, *B. anthracis* spores may possess special properties, which are absent in *B. subtilis*, that facilitate their persistence in the lung.

### Association of spores with the lung epithelium, epithelial cells, and phagocytes

**Figure 4:**
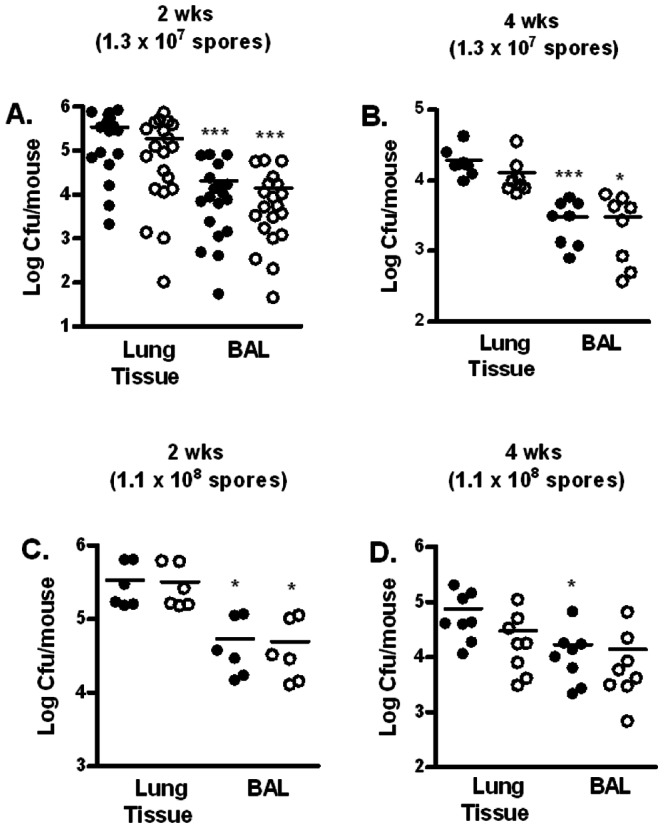
The majority of persisting spores associated tightly with the lung tissue. Mice were inoculated i.n. with ∼1.3×10^7^ spores/mouse (**A** and **B**) and ∼1.1×10^8^ spores/mouse (**C** and **D**). Lungs were lavaged with sterile PBS and collected. Total bacteria (closed circles) and spore (open circles) titers in the lung tissues and BAL fluids at 2 (**A** and **C**) and 4 (**B** and **D**) weeks were determined. The results were combined from at least two independent experiments. *, *p*<0.05; ***, *p*<0.001; compared to respective total bacteria and spore titers in the lung tissue.

We next examined whether the persistent *B. anthracis* spores were tightly associated with the lung tissue or in the fluid lining the respiratory epithelium, the latter of which can be recovered in the bronchoaveolar lavage (BAL) fluid. The results showed that at both 2 and 4 weeks post-inoculation, there were significantly more total bacteria as well as spores in the lung tissues than in the BAL fluid, suggesting that spores preferentially associated with lung tissues (Fig. 4, A–D).

**Figure 5:**
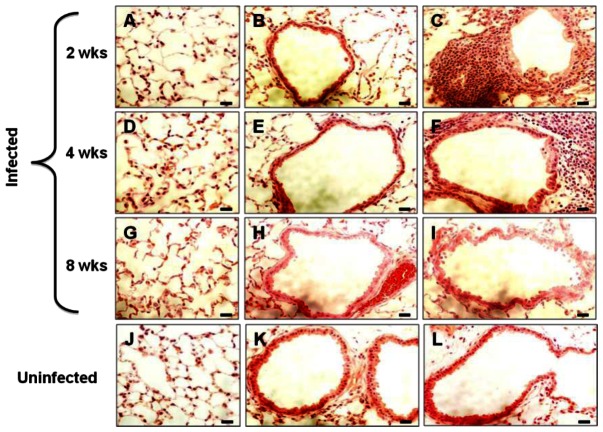
H&E stained lung sections of mice challenged with *B. anthracis* spores. Lungs from infected and control mice were collected at 2, 4, and 8 weeks post-inoculation, fixed and subjected to H&E staining. Representative images displaying the alveoli and the airway from mice infected for 2 (**A**–**C**), 4 (**D**–**F**), and 8 weeks (**G**–**I**), and uninfected control mice (**J**–**L**) are shown. Scale bars represent 20 µm.

We further analyzed lung sections from infected mice to determine the location of spores in the lung. Lung sections were obtained from mice infected with *B. anthracis* spores at 2, 4, and 8 weeks. Hematoxylin and eosin (H&E) staining of the sections showed minimal pathology in the lung (Fig. 5). The alveolar and small airway epithelium as well as blood vessels appeared intact. For most part of the lung, we observed no signs of inflammation (Fig. 5, A, B, D, E, G and H). We occasionally observed isolated foci that had inflammatory infiltrates at both 2 and 4 weeks (Fig. 5, C and F). At 8 weeks, the lungs were indistinguishable from the uninfected lung (Fig. 5, G–L).

**Figure 6:**
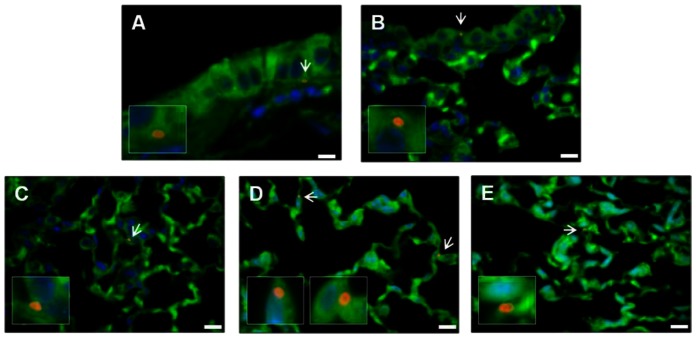
Representative images of immunofluorescently stained lung sections from mice challenged with *B. anthracis* spores. Mice were infected i.n. with ∼10^8^ spores/mouse. Lungs were harvested at 2 and 4 weeks, fixed, sectioned and stained with anti-BclA antibodies and secondary antibodies conjugated to Alexa Fluor 594 (red), Alexa Fluor 488-conjugated phalliodin (green) and DAPI (blue), as described in the Materials and Methods section. Representative images are shown to indicate spore association with the small airway epithelium (**A** and **B**) and the alveolar epithelium (**C**–**E**). Arrows indicate spores. The areas around those spores were enlarged and shown in boxed insets. Scale bars represent 10 µm.

**Figure 7:**
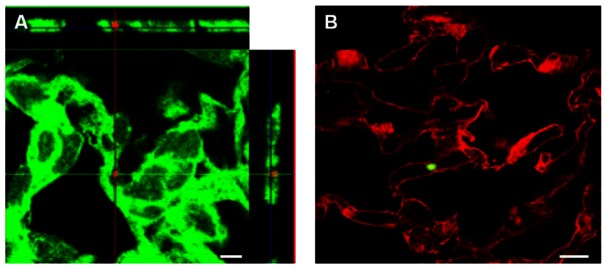
Confocal analysis of immunofluorescently stained lung sections. Lung sections were stained as described in the Materials and Methods section and analyzed by confocal microscopy. Representative images are shown. **A**, an image showing a spore (red) surrounded by F-actin (green). The projections show all planar views, including *xy* (center panel), *xz* (right panel) and *yz* (top panel)-stacks. **B**, the section was stained with wheat germ agglutinin to outline the plasma membrane (red) and anti-BclA antibodies to detect spores (green). Scale bars represent 10 µm.

To detect spores in the lung, immunohistochemistry was performed. Microscopic examination of the stained sections revealed that spores were present in the lung at all three time points. Spores were observed associated with the alveolar and the small airway epithelium as single spores rather than in clusters (Fig. 6, A–E), indicating that they did not form biofilms in the lung. Additionally, spores were seen distributed relatively evenly throughout the lung tissue similar to what was reported previously [13], [14]. Some of the spores that associated with the airway and alveolar epithelium appeared intracellular. To determine if the latter group of spores were indeed intracellular, we analyzed the Z-stacks of confocal images of 124 potential intracellular spores from 2 and 4 weeks. Of these, 101 (81%) were surrounded by F-actin (Fig. 7, A) or enclosed within the plasma membrane (Fig. 7, B) and therefore were likely to be intracellular.

**Figure 8:**
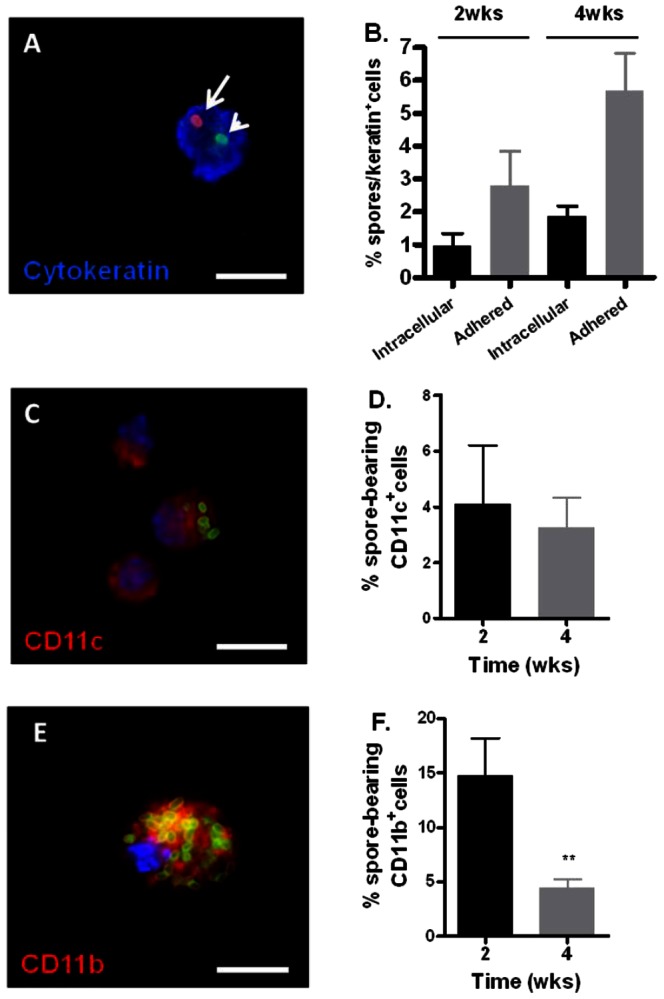
Spore association with epithelial cells and phagocytes in the lung. Mice were inoculated i.n. with ∼1.1×10^8^ spores per mouse. At 2 and 4 weeks post-infection, lungs were harvested and digested to obtain CLCS. Lungs were also lavaged to obtain BAL fluid. **A**, a representative image of a lung epithelial cell stained with anti-cytokeratin antibodies (blue) containing an extracellular spore (red, long arrow) and an intracellular one (green, short arrow). **B**, the percentage ratio of intracellular and extracellular adhered spores relative to the number of cytokeratin^+^ cells counted at 2 and 4 weeks post-inoculation, expressed as the mean±standard error of the mean (SEM). Approximately 500–600 cytokeratin^+^ cells were counted per mouse. **C** and **E**, representative images of cells stained positive with CD11c antibodies (**C**, red) and CD11b antibodies (**E**, red). Spores were stained green. Blue indicates DAPI staining for the nuclei. **D** and **F**, the percentage ratio of spore-containing CD11c^+^ and CD11b^+^ cells relative to the total number of CD11c^+^ and CD11b^+^ cells counted at 2 and 4 weeks post-inoculation, respectively, expressed as the mean±SEM. Approximately 100 CD11c^+^ cells and 500–600 CD11b^+^ cells were counted per mouse. Scale bars represent 10 µm. **, *p*<0.01 comparison between 2 to 4 weeks % spore-bearing CD11b^+^ cells.

To investigate the specific cell types with which spores associated, crude lung cell suspensions (CLCS) were prepared from lungs harvested at 2 and 4 weeks post-inoculation. Epithelial cells in the CLCS were detected using anti-cytokeratin antibodies (Fig. 8, A). Approximately 23% of the CLCS cells were cytokeratin-positive, consistent with previous results [13]. Approximately 3.8% and 7.6% of epithelial cells examined were associated with spores at 2 and 4 weeks, respectively. On average, the percentage ratio of intracellular and extracellular adhered spores relative to cytokeratin positive epithelial cells was 0.95±0.4% and 2.78±1.1% at 2 weeks, and 1.9±0.3% and 5.7±1.1% at 4 weeks, respectively (Fig. 8, B). Thus, spores associated with lung epithelial cells both extracellularly and intracellularly with the majority being extracellular. The CLCS also contained lung dendritic cells and residual alveolar macrophages that were not removed by lavage. To determine spore association with these phagocytes we stained the CLCS using anti-CD11c antibodies. Multiple spores were often seen inside these phagocytes (Fig. 8, C). Approximately 4.1% and 3.3% of CD11c^+^ cells contained spores at 2 and 4 weeks, respectively (Fig. 8, D). We also stained the BAL fluids with anti-CD11b antibodies and observed approximately 14.8% and 4.5% of CD11b^+^ cells contained spores at 2 and 4 weeks, respectively (Fig. 8, E and F). Many of the phagocytes appeared to be packed with spores however, making accurate enumeration of the intracellular spores difficult.

### The anthrax toxins are not involved in spore persistence in the lung

**Figure 9:**
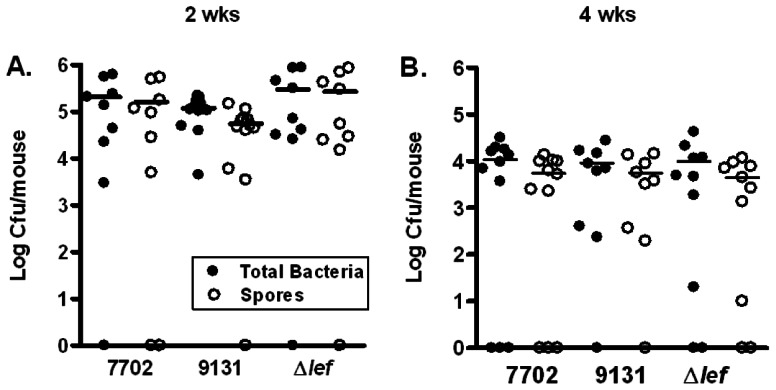
Bacterial and spore burden in the lungs of mice infected with toxin-deficient *B. anthracis* mutants. Mice were inoculated i.n. with ∼1.5×10^7^ spores. Lungs were harvested at 2 and 4 weeks post inoculation, homogenized and plated with or without heat treatment. The results were combined from two independent experiments. Closed circles represent total viable bacteria and open circles heat-resistant dormant spores.

Minimal inflammation observed in the lung from the H&E stained sections suggested that there was a subdued immune response in the presence of spores. *B. anthracis* lethal factor (LF) is a Zn^2+^-dependent metalloprotease that cleaves mitogen-activated protein kinase kinases (MEKs) and plays an important role in suppressing the host immune responses during anthrax infections [20]. We observed a low level of spore germination in the lung during the studies described above. LF was reported to be secreted shortly after spores germinate [21]. We investigated the possibility that LF secreted by the germinated spores suppressed the immune response and contributed to spore persistence in the lung. We compared the persistence of spores from the Sterne strain 7702 with that from the isogenic LF-deficient strain (Δ*lef*) [22] as well as a plasmid-free Sterne strain 9131, which does not produce any anthrax toxins. We did not observe any significant difference in either the total bacteria or the spore counts between the three strains at 2 or 4 weeks post-inoculation (Fig. 9, A and B), indicating that the anthrax toxins do not play a role in the persistence of spores in the mouse lung.

## Discussion

Recovery of *B. anthracis* spores from the lungs of animal hosts weeks or months after the initial pulmonary exposure has been known for decades and is the basis for the recommended 60-day antibiotic regimen for people with pulmonary exposure to spores [2], [3], [4]. However, beyond this knowledge detailed information on how spores persist *in vivo* and factors contributing to persistence was lacking. Here we investigated the spatial distribution of spores in mice and the association of spores with different types of host cells for up to 8 weeks. We also compared the persistence between *B. anthracis* spores and *B. subtilis* spores. The results provided insights into the potential mechanisms underlying spore persistence, as discussed below.

It has been reported that during late stages of acute inhalational anthrax infections large amounts of vegetative bacilli of *B. anthracis* were present in multiple organs including the liver, kidney, lymphoid tissues, and even the brain, presumably via hematogenous spread [3], [19]. Results from the current study suggest that chronic persistence of spores *in vivo* exhibits a different pattern of tissue distribution. The lung is the primary site for spore persistence. We also recovered heat-sensitive bacilli in the lung, but at significantly lower amounts compared to spore titers. These results combined with positive staining of lung sections with antibodies specific for vegetative bacilli suggest that there may be a low level of spore germination in the lung during persistent infections. It is possible that germination may occur inside host cells as a number of previous studies suggested intracellular germination using *in vitro* cultured cells [13], [14], [23], [24]. The possibility that spores germinate extracellularly in the lung at a low frequency cannot be excluded either. It is possible that a relatively large bolus of spores may cause damage to the lungs, providing germinants not normally present due to spore abrasion as suggested by Glomski *et al* [19]. In acute infections, the site of spore germination after pulmonary exposure has been investigated by different groups. Findings by Glomski *et al* [19] and Ross [25] indicated that spore germination occurred in organs other than the lung. However, studies by Sanz *et al* utilizing a Sterne strain that specifically expressed bioluminescence only upon germination reported germination proceeded in the lung [26]. The use of different bacterial/mouse strains experimental methodologies and/or different spore doses could have contributed to the different conclusions [19]. We also observed some spores in the other organs examined; however, the titers are orders of magnitude lower than those in the lung. These spores presumably spread to the distal organs via phagocytic cells.

The results also show that the majority of persistent *B. anthracis* spores appeared to be tightly associated with lung tissues, as they could not be readily removed by lavage. Further examination of thin sections of the lung revealed that the spores adhered to the alveolar and airway epithelium and some were inside the cells lining the epithelium. This was further corroborated by immunofluorescence staining of lung cells harvested from the infected lungs. Differential immunofluorescence staining of lung epithelial cells indicated that there were more extracellularly adhered spores than intracellular ones. This suggests that adherence to the lung epithelium may be an important mechanism for spores to persist in the lung. Residing in an intracellular niche of lung epithelial cells may be a secondary mechanism. The results also indicated that epithelial cells were not the only cell type that spores associated with. We observed spores inside CD11b^+^ and CD11c^+^ cells at both 2 and 4 weeks, and most of the spores appeared intact. The results in Fig. 4 indicated that viable spores were recovered from the BAL fluids at 2 and 4 weeks. These results suggest that spores may be viable inside the phagocytes, consistent with previous reports that spores were able to survive inside phagocytic cells [27]. Therefore, it is possible that the intracellular environment of phagocytic cells is a niche for spore persistence. On the other hand, the fate of phagocytosed *B. anthracis* spores has been investigated by multiple groups with somewhat controversial results. Spores are thought to germinate inside macrophages. Some of the studies reported replication of vegetative bacilli inside macrophages [21], [24], [28], while others showed killing of germinated spores and vegetative bacilli by macrophages [29], [30], [31]. Therefore, recovery of viable spores from phagocytes does not exclude the possibility of phagocytic killing of germinated spores and vegetative bacilli. Also, it is possible that the amount of spores has an impact on the fate of *B. anthracis* within macrophages [30], [32]. The finding that total bacteria and spore titers in the lung significantly decreased over the experimental period, while the titers in other organs examined remained very low suggested a continuous host clearance process. Therefore, it is also possible that the spore-bearing CD11b^+^ and CD11C^+^ cells we observed reflect a part of the clearance process.

We investigated if the ability to persist in the lung could be a general phenomenon of *Bacillus* spores, *i.e.*, if spores from other species of *Bacillus* could also persist in the lung. The significantly lower bacterial/spore burden in the lungs of mice exposed to spores of a *B. subtilis* strain suggests that *B. anthracis* spores possess special properties that promote their survival and persistence in the lung. *B. subtilis* spores are known to share a similar coat protein profile as that of *B. anthracis* spores but lack an exosporium which is present on the latter [33]. The exosporium is the outermost integument of *B. anthracis* spores and is composed of 20–30 proteins and glycoproteins [34], [35], [36]. It is possible that specific exosporium components contribute to *B. anthracis* spore persistence in the lung. It was previously shown that surface components on *B. anthracis* spores were sufficient to mediate spore adherence and entry into epithelial cells [14] and that *B. subtilis* spores were much poorer at adherence or entry into host cells [37]. It was reported previously that entry of *B. anthracis* spores into epithelial cells was mediated by the spore surface glycoprotein BclA and host cell receptor integrin α2β1 via a novel mechanism that requires complement component C1q as a bridging molecule [38]. Spores from a BclA deletion mutant (*ΔbclA*) showed decreased entry into epithelial cells compared to spores from the isogenic parent strain [38]. However, other studies reported a different role for BclA that it reduces spore entry into non-phagocytic host cells [39], [40]. One of the factors contributing to the discrepancy may be the germination status of spores in the assays. It seems that if spores remain dormant, BclA plays an important role in host cell entry via the BclA-C1q-α2β1 pathway. However, if spores are allowed to germinate in the assay media, having BclA appeared to be a disadvantage in host cell entry [38]. As the majority of spores remain dormant in the host lung, results from dormant spores are likely to be more relevant to the *in vivo* situation. Studies to investigate the role of BclA in spore persistence *in vivo* is currently underway in our laboratory.

Evans *et al*. reported that a bacterial lysate could induce innate resistance to infections caused by pulmonary exposure to *B. anthracis* spores. The authors further demonstrated that lung epithelial cells rather than macrophages or neutrophils were responsible for the induced resistance [41]. This demonstrates that the lung epithelium is an important player in the host defense against infections by *B. anthracis* spores. It is conceivable that in addition to mediating adherence and entry, interactions between exosporium components and the lung epithelium may also influence the host immune responses in a way that favors the survival of spores in the lung. We are currently carrying out studies to investigate the precise role of specific exosporium components in spore persistence.

The very mild inflammation and pathology observed in the lung is also indicative of a subdued immune reaction to *B. anthracis* spores. While the spore surface does not have any typical pathogen-associated molecular patterns such as LPS, LTA, peptidoglycan, or flagellin, a number of studies indicated that *B. anthracis* spores are not “stealth” to host immune recognition. Spores are capable of activating Toll-like receptor 2 and MyD88-dependent signaling [42], inducing inflammatory cytokine production in both MyD88-dependent and independent manners [43], [44], activating natural killer cells [45], [46] and initiating the classical complement pathway via a direct interaction between the exosporium component BclA and C1q [47]. These findings from the literature suggest that the subdued immune response may be due to an active immune evasion/suppression mechanism of *B. anthracis* rather than passive inactivity of the spores. We demonstrated that the anthrax toxins, in particular the lethal factor, a major immune suppressor of *B. anthracis*, did not play a role in spore persistence in the lung. This implies that *B. anthracis* exosporium components may have immune suppression properties. We are currently investigating this possibility.

In summary, the results described in this study suggest that there are likely multiple mechanisms contributing to spore persistence in the mouse lung. Association with the lung epithelium and immune suppression/evasion are two potential mechanisms. The results also suggest that spore surface components play important roles in mediating persistence. The work presented here has provided a foundation for further studies to elucidate the molecular mechanisms responsible for spore persistence. Understanding the mechanisms of persistence may potentially provide clues for developing more effective therapeutic regimens for anthrax infections and have implications for other persistent or chronic infections in general.

## Materials and Methods

### Bacterial strains and spore preparation

*B. anthracis* Sterne strain 7702, its isogenic toxin-deficient mutant strain (*Δlef*) [22], plasmid-free *B. anthracis* strain 9131 derived from Sterne Strain 7702, and *B. subtilis* strain PY79 were provided by T. M. Koehler, Univ. of Texas, Houston, TX. Spores of these strains were prepared by culturing in PA media for 10 days at 30°C as described previously [13]. *E. coli* HB101 was grown in Luria Broth overnight at 37°C with shaking. The overnight cultures were washed three times with sterile PBS prior to inoculation into mice. Bacterial counts were determined by plating on Luria Broth agar plates and incubation overnight at 37°C.

### Mouse infections

All animal experiments were carried out according to procedures approved by the Institutional Animal Care and Use Committee, Texas A&M Health Science Center, Institute of Biosciences and Technology (IBT). BALB/c mice were originally purchased from the Jackson Laboratory and maintained in the IBT animal facility. For infection experiments, 6–8 weeks old and sex-matched mice were used. Mice were inoculated with spores or *E. coli* by intranasal instillation (i.n.) as previously described [48]. Briefly, mice were anesthetized with avertin (0.3 mg/g) by intraperitoneal injection (i.p.). Mice were then inoculated i.n. with 20 µls of a sub-lethal dose of spores (LD_50_ was determined in pilot studies to be ∼1.7×10^8^ spores/mouse). Mice were monitored twice daily. Mice mostly appeared healthy with no physical signs of distress or illness throughout the experiments. To determine the bacterial burden in various organs mice were euthanized by i.p. injection of avertin followed by exsanguination via the inferior cava vein. Lungs, kidneys, spleen, trachea and nasopharynx were collected at indicated time points. The tissues were homogenized in sterile cold PBS plus D-alanine, pH 7.4 (1 ml final volume) using a tissue homogenizer (Fisher Scientific). BAL fluids were collected by lavaging the lungs with sterile PBS twice. The homogenates and BAL fluids were either directly diluted and plated to determine the number of total viable bacteria or heated at 68°C for 1 hr and dilution plated to determine the number of heat-resistant spores.

### Isolation and immunofluorescence staining of crude lung cell suspension (CLCS)

Lungs were harvested from mice at indicated time points and digested as previously described to obtain crude lung cells [13]. The CLCS was stained using a previously described procedure [13] with some modifications. To stain for specific cell types and spores, cells in the CLCS were allowed to attach to poly-L-lysine coated coverslips and then blocked with PBS containing 5% goat serum. Anti-BclA antibodies (rabbit polyclonal antibodies raised against recombinant BclA, a spore surface protein, 1∶100) was added to the cells and incubated for 2 hours followed by secondary antibodies conjugated to Alexa Fluor 594 to detect extracellular spores. Cells were then permeabilized, blocked and incubated again with anti-BclA antibodies followed by secondary antibodies conjugated to Alexa Fluor 488 to detect both extracellular and intracellular spores. Epithelial cells were detected with a pan epithelial mouse monoclonal antibody (Clone C-11, Chemicon, 1∶250) recognizing different isoforms (4, 5, 6, 8, 10, 13, and 18) of cytokeratin, an epithelial specific marker, followed by secondary antibodies conjugated to Alexa Fluor 350. Anti-CD11c and anti-CD11b antibodies (Caltag Laboratories, 1∶250) were used to stain phagocytes in the CLCS and BAL fluids, respectively. Coverslips were mounted on slides using Fluorsave (Calbiochem) and viewed with a Zeiss Axiovert 135 microscope with the Axio Vision Software.

### Histology and immunofluorescence staining of lung sections

This was done following a previously described procedure with slight modifications [13]. Briefly, lungs were harvested at indicated time points and fixed in 10% formalin. Embedding, sectioning and H&E staining were performed at the Breast Center Pathology Laboratory, Baylor College of Medicine, Houston, TX. To detect spores, lung sections were incubated in a solution containing anti-BclA antibodies (1∶250) for 2.5 hours followed by incubation with Alexa Fluor 594-conjugated secondary antibodies for 1 hr. Phalloidin Alexa Fluor 488 was used to stain F-actin. DAPI was used to stain the nuclei. Some lung sections were also stained with wheat germ agglutinin Texas Red 595 (Invitrogen, 1∶1000) to detect the plasma membrane. All dilutions were made in PBS pH 7.4 containing 2.5% fetal bovine serum. Five slides per mouse at each time point were examined. Lung sections were viewed with a Zeiss Axiovert 135 fluorescence microscope with the Axio Vision Software and an LSM 510 confocal laser scanning fluorescent microscope with the LSM 4.0 software (Zeiss).

### Statistical analysis

Statistical analysis was performed using the two-tailed Student's *t*-test (Graph-Pad Prism 4.0).

## Supporting Information

Figure S1 **Detection of vegetative bacilli in the lung by immunofluorescence staining.** **A**, Antibodies against BclA and CW7 are specific for spores and vegetative bacilli, respectively. Spores of *B. anthracis* Sterne strain 7702 were incubated in DMEM, 10% FBS for 0hr (spores) and 3hrs (vegetative bacilli). Bacteria were then spun onto poly-L-lysine coated coverslips and subjected to immunofluorescence staining. Antibodies against BclA and CW7, a cell wall anchored protein of *B. anthracis*, were rabbit antibodies raised against purified recombinant proteins of BclA and CW7. Secondary antibodies were goat anti-rabbit IgG–Alexa Fluor 633. Spores were pre-labeled with FITC for visualization. Fluorescence from FITC was barely visible after 3hrs of incubation; therefore, vegetative bacilli were visualized by staining their nuclei with DAPI. **B**, Detection of vegetative bacilli using anti-CW7 antibodies. A representative image of positive CW7 staining of a lung section from a mouse lung harvested at 2 weeks post-inoculation. Lung sections were fixed, sectioned, and stained with rabbit anti-CW7 antibodies and secondary antibodies conjugated to Alexa Fluor 594 (red), Alexa Fluor 488-conjugated phalliodin (green) and DAPI (blue). Scale bar represents 10 µm.
(TIF)

## Acknowledgments

We thank Gabriela Bowden, Texas A&M Health Science Center-IBT, Houston, TX, and Laura Kim, Department of Pulmonary Medicine, M. D. Anderson Cancer Center, Houston, TX for help with mouse infection experiments and for harvesting the NALT from mice. We thank T. M. Koehler, University of Texas Medical School, Houston, TX, for providing *B. anthracis* and *B. subtilis* strains. We also thank Chunfang Gu, Cleveland Clinics, Cleveland, OH, for technical assistance.
