# Supplementary figures and images for: Characterization of Bacillus anthracis Persistence In Vivo
Source: PLoS One. 2013 Jun 4;8(6):e66177. doi: 10.1371/journal.pone.0066177 (PMC3672131; doi:10.1371/journal.pone.0066177)

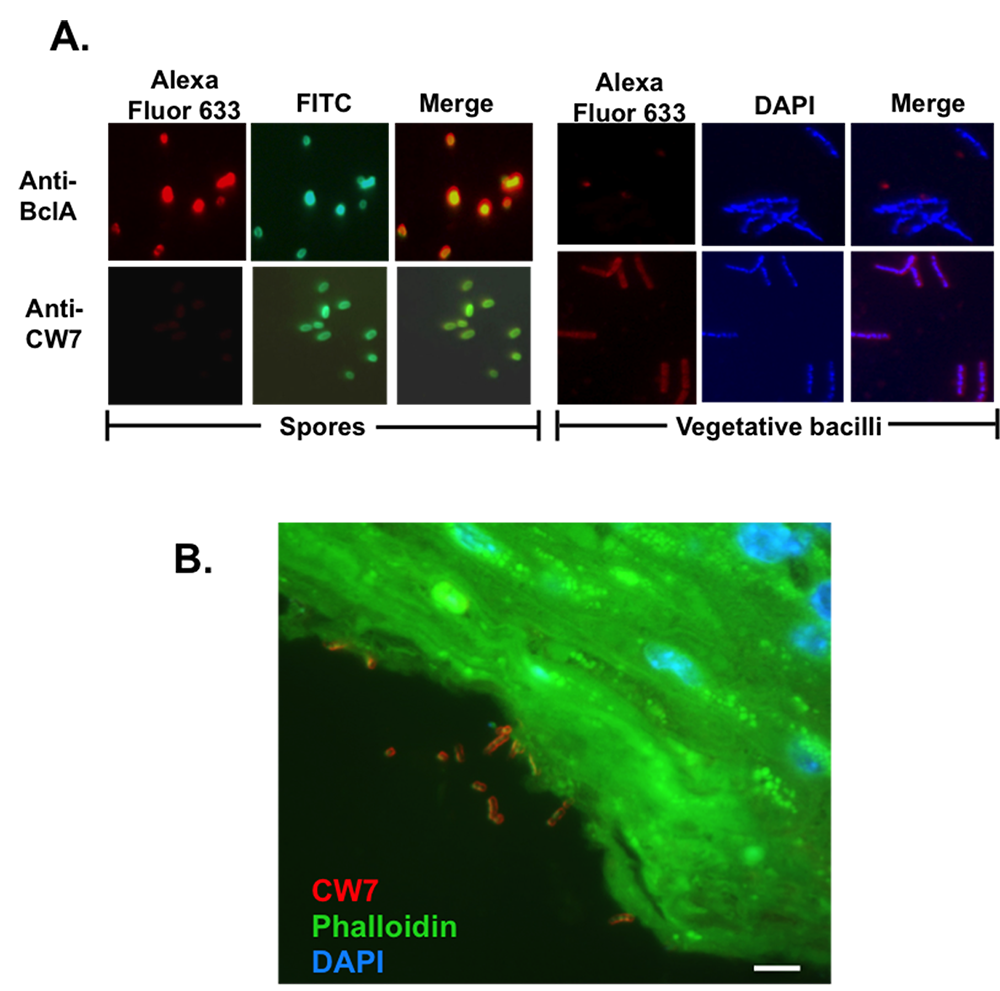

Supplement: Figure S1 — Detection of vegetative bacilli in the lung by immunofluorescence staining. A, Antibodies against BclA and CW7 are specific for spores and vegetative bacilli, respectively. Spores of B. anthracis Sterne strain 7702 were incubated in DMEM, 10% FBS for 0hr (spores) and 3hrs (vegetative bacilli). Bacteria were then spun onto poly-L-lysine coated coverslips and subjected to immunofluorescence staining. Antibodies against BclA and CW7, a cell wall anchored protein of B. anthracis, were rabbit antibodies raised against purified recombinant proteins of BclA and CW7. Secondary antibodies were goat anti-rabbit IgG–Alexa Fluor 633. Spores were pre-labeled with FITC for visualization. Fluorescence from FITC was barely visible after 3hrs of incubation; therefore, vegetative bacilli were visualized by staining their nuclei with DAPI. B, Detection of vegetative bacilli using anti-CW7 antibodies. A representative image of positive CW7 staining of a lung section from a mouse lung harvested at 2 weeks post-inoculation. Lung sections were fixed, sectioned, and stained with rabbit anti-CW7 antibodies and secondary antibodies conjugated to Alexa Fluor 594 (red), Alexa Fluor 488-conjugated phalliodin (green) and DAPI (blue). Scale bar represents 10 µm. (TIF) [file pone.0066177.s001.tif]
